# Editorial: Environmental contaminants in aquatic systems and chemical safety for environmental and human health, volume II

**DOI:** 10.3389/fpubh.2023.1157834

**Published:** 2023-06-13

**Authors:** Mohiuddin Md. Taimur Khan, Larry Sklar

**Affiliations:** ^1^Department of Civil and Environmental Engineering, Washington State University Tri-Cities, Richland, WA, United States; ^2^Center for Molecular Discovery and Cancer Center, University of New Mexico, Albuquerque, NM, United States

**Keywords:** environmental contaminants, chemical safety, aquatic system pollution, environmental and human health, carcinogenicity, mutagenicity, ambient water quality

Given the finite supply of water available for human use, the continued chemical contamination of the aquatic environment may pose a significant human health hazard. Consequently, an effort must be made to develop ambient water quality criteria to protect human health and preserve the integrity of the aquatic environment. In developing water quality criteria based on human health effects, information on sources of exposure, pharmacokinetics, and adverse effects must be carefully evaluated and acknowledged. Information and fundamental knowledge on the sources of exposure are needed to determine the contribution of exposure from water relative to all other sources.

Human exposure to hazardous agents in our food, air, and water contributes to illness, disability, and death. Poor environmental quality has its greatest impact on people whose health may already be at risk, notably, pregnant women, young children, older adults, and people with preexisting illnesses. National efforts to ensure clean and safe food and water supplies continue to contribute significantly to improvements in public health and the prevention of disability. Currently, carcinogenicity and mutagenicity are considered to be non-threshold effects. For carcinogens and mutagens, criteria are calculated by postulating an “acceptable” increased level of risk and using extrapolation models to estimate the dose which would result in this increased level of risk. For other chemicals, thresholds are assumed, and criteria are calculated by deriving “acceptable daily intakes” for man which would presumably result in no observable adverse effects.

In recent years, antidepressants have acquired much attention because of their occurrence in water from the environment and aquatic organisms, as well as their potential harm to ecosystems and human wellbeing. The toxicological effects of antidepressants in different organisms, primarily fish, aquatic plants, and mammals included changes in weight, pathological changes in the brain, heart, and kidney, and a decrease in sperm dose (1, 2). It is also known that art materials may contain chemicals, which are associated with chronic toxicity (3, 4). Some of these chemicals include heavy metals such as nickel chloride that can potentially dysregulate mechanisms involved in genome maintenance and repair (5) and may predispose human cells to oncogenesis.

Recent scientific studies have demonstrated that insecticides have a strong collateral effect on both human and other non-target organisms and often on pests. Furthermore, the brown planthoppers (a serious rice pest) outbreak can be traced to the misuse of insecticides. Current pest management solely depends on chemical pesticides with effects on the environment, biodiversity, and human health (6). Although much progress has been made, crayons are among the most widely used products by children and can potentially be contaminated with lead, and there is a great need to further minimize the exposure to ensure the safety of consumers (7).

The risk for carcinogenic and non-carcinogenic effects associated with the exposure to contaminants through three specific mechanisms (e.g., water pollution, food adulteration, and biomagnification) can be variable depending on the types of contaminants, their respective properties, and natural attenuation or digestive mechanisms. Often, these contaminants become the part of food chain due to poor control of effluent treatment plants of textiles, tanneries, and pharmaceuticals industries as well as the open dumping of toxic/solid waste and wastewater (8, 9). Heavy metals including cadmium, mercury, lead, copper, and zinc are recognized as important marine pollutants because of their toxicity, presence in food chains, and propensity to survive in the environment for an extended period (10, 11). Leather manufacturing involves many chemical products such as chromium sulfate, tannins, bactericides, and ammonia salt (12). Moreover, protecting the shellfish aquaculture farms often requires the prevention of oyster consumption when bacterial levels are high in water (13–16).

Researchers have identified that the wastewater treatment plants were primary sources of emerging contaminants (ECs) observed in surface water samples (17). The prominent classes of ECs mostly include pharmaceuticals and personal care products (PPCPs), nanomaterials, surfactants, heavy metals, fire retardants, plasticizers, fertilizers, and pesticides (18, 19). Several classes of the ECs were recognized as endocrine disruptive compounds (EDCs) due to their deleterious effects on endocrine systems (EDCs). The impact of ECs has been reported in surface water, wastewater, and groundwater sources (18, 19). Effluents from the pharmaceutical industry are another important source, with high concentrations of pharmaceuticals being found due to discharges from factories in several parts of the world despite strict regulation of of pharmaceutical products (20–24). The ECs can effectively be eliminated by up to 99%, using the membrane bioreactor (MBR) and advanced treatment technologies such as reverse osmosis, ultrafiltration, or nanofiltration (25). The tertiary treated wastewater is discharged into the open water sources after meeting the water quality standards and is not used as a palatable source of water. Therefore, wastewater treatment plants do not use MBR technologies, which are not energy-efficient and cost-effective. Therefore, it is erroneous to assume that the traditional tertiary treated wastewater is free of these emerging contaminants (26–28). Petrie et al. (29) confirmed that wastewater treatment procedures used in the treatment plants were not effective in completely removing emerging contaminants.

Transport pathways of heavy metals and other ECs from the soil into the aquatic ecosystems are a major concern in pollution and contamination because they depend on the solubility of ECs and are influenced by aerobic or anaerobic conditions, pH, and redox potential (30). These ECs not only impair soil quality and freshwater sources but could also get into the food chain and affect human and animal health, i.e., one health. Metal type and their bio availabilities in soils determine the extent of physiological uptake and potential toxic effects of metals in living organisms (31). On the other hand, antibiotic-resistant bacteria are resistant to both natural and synthetic antibiotics (32) and thus have become a health concern worldwide. Multi-drug resistant bacteria (MDRB) with stronger resistance can be resistant to three or more antibiotics in the clinic (33, 34). Bacteria can develop intrinsic resistance to certain antibiotics but can also acquire resistance to antibiotics (35). The pathway for bacteria to acquire or develop antibiotic resistance, which is rooted in the irrational usage of antibiotics, is to prevent antibiotics from entering the target, change the antibiotic targets, and inactivate antibiotics (36, 37). The irrational usage of antibiotics can lead to the prolonged exposure of bacteria to sublethal concentrations of antibiotics, which is key to resistance selection (38).

Only a small portion of the antibiotics in aquatic products are actually absorbed, with most being discharged into the environment, resulting in antibiotic residues in aquaculture areas in discharged wastewaters and accumulated in the surrounding sediments through adsorption (39, 40). In livestock farming, antibiotics are important for the prevention of infectious diseases and their treatment as well as for promoting the growth of livestock (41). Antibiotics applied to livestock and poultry are not fully absorbed, with most being excreted into the environment through animal feces or urine (42).

Based on the above discussions, there could be strong correlations among the micropollutants, metals, harmful chemicals, ECs, antibiotics, microbes, and aquatic environmental agents, which have an effect on the public health, food chain, soil-water environments, and animals—the major parameters of one health (Figure 1). Majed et al. (43) discussed the influence of contaminant pathway to water and soil on hygiene and healthy habits, which is a behavior parameter. However, conservation habits can help conserve water, increase food supply, and provide shelter for animals, birds, and insects. These habits are consistent with actions helping to protect and manage natural resources. Many of those habits will help establish and maintain healthy habitats, which are flourishing places for animals and others to live. Furthermore, these habitats provide a strong foundation for the ecosystem toward sustainable public health policy, resilience to withstand change and stressors, and solutions for climate change. Recent evidence from European ice cores showed a strong relationship between unusual weather (low temperatures and high rainfall) and the severity of the Spanish Flu epidemic during the First World War (44). There is evidence that Hg and persistent organic pollutants (POPs) removed from the atmosphere and deposited on snow have been released to the environment at snowmelt, rapidly dispersing hazardous compounds through the atmosphere, continental, and aquatic systems and becoming bioavailable to be incorporated into food webs (45, 46).

**Figure 1:**
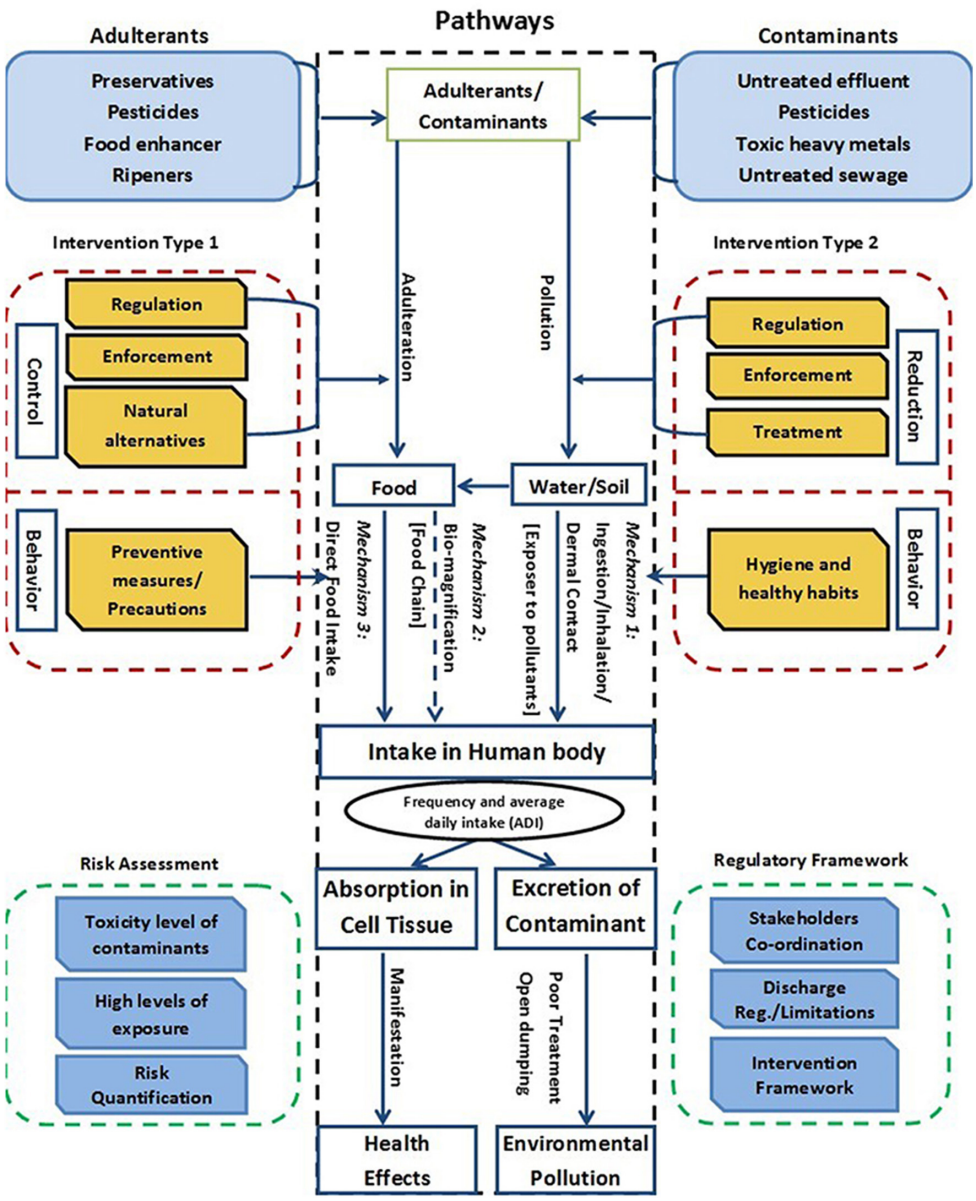
Flow chart of human exposure to contaminant pathways and associated mechanisms involved and framework for interventions (43).

Climate change affects the frequencies and durations of viral epidemics by altering the distribution, abundance, and activity of hosts, changing resistance to infection, the physiology of host-virus interactions, the rate of virus evolution, and host adaptation (47, 48). According to the World Health Organization (49), solid fuel includes coal as well as biomass fuels (referring to renewable plant-based materials such as wood, crop wastes, and charcoal), providing heat and light during the process of combustion. Ambient air pollutants (e.g., particulate matter and polycyclic aromatic hydrocarbons) may cause tumor formation in the breast and cervix uteri (50–52).

It has long been known that exposure to high levels of certain chemicals, such as those in some occupational settings, can cause cancer. Cancer is the second leading cause of death in the United States; it accounts for one in four deaths in the US and claims more than 1,500 lives a day. There is now growing scientific evidence that exposure to lower levels of chemicals in the general environment is contributing to society's cancer burden and health hazard. It is eminent to adapt the emerging regulations, treatment technologies, public awareness, resource management, and policy assessment to overcome the environmental contaminants-related threat and issues in the environment. Moreover, chemical safety for environmental, animal, and human health is a mandatory concern, and proper management and regulations are necessary to adopt advanced and accurate safety measures.

## Author contributions

MK conceptualized the editorial article, performed the literature review, validation, and designed the article preparation. MK and LS finalized the editorial article. LS confirmed the format and requirements of this submission. Both authors contributed to the article and approved the submitted version.
